# 

**Published:** 1918-08-17

**Authors:** 


August 17th, 1918.] [Price Twopence

### The Hospital

The Workers' Newspaper of Administrative Medicine and Institutional Life, Administration, National Insurance and Health.

CONTENTS.

**Medical Education** ... ... ... ... ... 423

Sir George Newman Performs a Miracle.

**Is Small-Pox Imminent?** ... ... ... ... ... 429

Its Incidence during Recent Years.

**Medicine in America** ... ... ... ... ... 433

By Sir William Osler, F.R.S.

**Social Service at St. Thomas's Hospital** ... ... 428

**London's Hospital Sunday Yields £85,000** ... 424

**Hospital and Institutional News** ... ... ... 425
Her Majesty's Visit to Brighton—A Tour of Belfast Hospitals—Highbury as a Permanent Hospital—A Leicester Lady's Generous Bequests—A Canon in the Ring—Sectional Subscriptions at Edinburgh—Women Medical Students at University College Hospital—The Trench-Fever Problem—A Medical Officer on Education—Chinese Medical Women—A Seventy-seventh Annual Meeting—Accident Cases at Poor-Law Infirmaries—A Ward in a Workshop—A Convalescent Hospital Closed—Naming a Bed After a Secretary—Should Red Cross Hospitals be Rationed?—This Week's Drug Market.

CONTENTS.

**The Matrons', Sisters', and Women's Departments—**

**A Hospital's Nursing and Domestic Staff** ... ... 435

How to Organise Both.

**Round the Hospitals** ... ... ... ... ... 435

National Health: Health Visitors and Nurses—Hints with Criticism.

**A Nurses' Charter of Liberty** ... ... ... ... 434

Who Fears to Speak? By "Ierne."

**Queen Mary and the Legless** ... ... ... ... 425

**The Editor's Letter-Box** ... ... ... ... 437
Matron's and Nurses who Prefer the "London" Training—The Silence of the Nurse: One Suggested Reason—"Ierne's" Prodding Processes.

**The Young Girl in Industry** ... ... ... ... 431

Her Health, Common Dangers and Salvation.

**Parliament and the Profession** ... ... ... vi

**The Institutional Worker** ... ... ... ... I
Bedroom Suppers for Nurses Off Duty.

